# GENDER DISPARITIES IN WAGES AMONG OLDER WORKERS: THE ROLE OF OCCUPATIONAL SEGREGATION AND EDUCATION

**DOI:** 10.1093/geroni/igae098.1855

**Published:** 2024-12-31

**Authors:** Rebekah Carpenter

**Affiliations:** Florida State University, Tallahassee, Florida, United States

## Abstract

Women have a long history of being underpaid relative to men, and many argue that these differences are driven by the concentration of women in jobs that pay less, along with differences in skills and education. Despite increased educational attainment and labor market participation by women over the last 50 years, today, men continue to outearn women. One way to assess structural/contextual factors contributing to gender disparities in pay involves evaluating occupational sex segregation (i.e., the unequal distribution of men and women across different occupations and industries) and the extent to which college education leverages differing levels of financial remuneration among older men and women at the latest phases of their working lives, a sample often excluded from studies on gender pay disparities. Leveraging data from the American Community Survey (2015-2019), I examine the relationship between occupational sex segregation, college degree status, and gender disparities in earnings for a nationally representative sample of 2,101,547 older workers ages 50-70. Findings from regression analyses show that older women earn significantly less than older men across each occupational sex segregation category (i.e., male-dominated, gender-neutral, or female-dominated), regardless of college degree status. Moreover, the greatest disparities in earnings are among college-educated older workers in gender-neutral occupations, where women earn only 70 cents for every one-dollar men earn. Findings from this study highlight the structural/contextual factors shaping the basis of gender pay disparities in later phases of working careers, which has important implications for contextualizing gender differences in retirement preparation and later-life wealth and wellbeing.

